# A practical approach to pancreatic cancer immunotherapy using resected tumor lysate vaccines processed to express α-gal epitopes

**DOI:** 10.1371/journal.pone.0184901

**Published:** 2017-10-27

**Authors:** Kenta Furukawa, Masahiro Tanemura, Eiji Miyoshi, Hidetoshi Eguchi, Hiroaki Nagano, Katsuyoshi Matsunami, Satoshi Nagaoka, Daisaku Yamada, Tadafumi Asaoka, Takehiro Noda, Hiroshi Wada, Koichi Kawamoto, Kunihito Goto, Kiyomi Taniyama, Masaki Mori, Yuichiro Doki

**Affiliations:** 1 Department of Gastroenterological Surgery, Osaka Police Hospital, Osaka, Japan; 2 Department of Molecular Biochemistry and Clinical Investigation, Osaka University Graduate School of Medicine, Osaka, Japan; 3 Department of Surgery, Osaka University Graduate School of Medicine, Osaka, Japan; 4 Department of Gastroenterological, Breast and Endocrine Surgery, Yamaguchi University Graduate School of Medicine, Yamaguchi, Japan; 5 Department of Phamacognosy, Hiroshima University Graduate School of Biomedical and Health sciences, Hiroshima, Japan; 6 Institute for Clinical Research, National Hospital Organization Kure Medical Center and Chugoku Cancer Center, Kure, Japan; Vrije Universiteit Brussel, BELGIUM

## Abstract

**Objectives:**

Single-agent immunotherapy is ineffective against poorly immunogenic cancers, including pancreatic ductal adenocarcinoma (PDAC). The aims of this study were to demonstrate the feasibility of production of novel autologous tumor lysate vaccines from resected PDAC tumors, and verify vaccine safety and efficacy.

**Methods:**

Fresh surgically resected tumors obtained from human patients were processed to enzymatically synthesize α-gal epitopes on the carbohydrate chains of membrane glycoproteins. Processed membranes were analyzed for the expression of α-gal epitopes and the binding of anti-Gal, and vaccine efficacy was assessed *in vitro* and *in vivo*.

**Results:**

Effective synthesis of α-gal epitopes was demonstrated after processing of PDAC tumor lysates from 10 different patients, and tumor lysates readily bound an anti-Gal monoclonal antibody. α-gal(+) PDAC tumor lysate vaccines elicited strong antibody production against multiple tumor-associated antigens and activated multiple tumor-specific T cells. The lysate vaccines stimulated a robust immune response in animal models, resulting in tumor suppression and a significant improvement in survival without any adverse events.

**Conclusions:**

Our data suggest that α-gal(+) PDAC tumor lysate vaccination may be a practical and effective new immunotherapeutic approach for treating pancreatic cancer.

## Introduction

Despite the general trend towards increased cancer survival, the prognosis for pancreatic ductal adenocarcinoma (PDAC) patients remains poor. Although PDAC is currently fourth on the list of leading causes of cancer-related mortality, the disease is predicted to rise to second by 2030 in Western countries.[1] This is related to the fact that PDAC is asymptomatic until the sudden appearance of prominent clinical symptoms and signs of advanced disease; despite surgery, and locoregional chemo- and molecular therapies, the overall median survival is less than 1 year from diagnosis.[2,3] While surgical resection provides the best opportunity for a cure, only approximately 20% of patients have resectable or borderline resectable disease, and are thus candidates for potentially curative surgery with neoadjuvant or adjuvant treatment.[4] The median survival for patients with unresectable metastatic PDAC remains less than 1 year, and the 5-year overall survival rate for PDAC is only 6%.[5]

The search for new therapies has mainly focused on the improvement of radio/chemo treatments, and to date there are only a few chemotherapeutic agents that show promise against PDAC, including gemcitabine with or without nab-paclitaxel[2] as well as the combination of 5-FU, leucovorin, oxaliplatin and irinotecan (so-called FOLFIRINOX regimen).[3] Despite this, the improvement in survival has been marginal over the past three decades, and there is an urgent need for novel and more effective therapeutic approaches for the treatment of PDAC.

Cancer immunotherapy is a potential paradigm shift for the treatment of PDAC patients. Ipilimumab, a monoclonal antibody (mAb) that blocks the immune checkpoint cytotoxic T lymphocyte antigen-4, was approved by the United States Food and Drug Administration in 2011.[6] More recently, other checkpoint inhibitors, including programmed-death-1 and programmed-death-1 ligand-1 blocking antibodies (Abs), were shown to induce objective responses in approximately 20–30% of patients with various cancers, including melanoma, non-small cell lung cancer, and renal cell carcinoma.[7,8] Despite the success of single-agent immunotherapy for some cancers, this approach has been unsuccessful in the treatment of PDAC. One difference between PDAC and cancers responsive to single-agent immunotherapies is the immune status of the tumor microenvironment (TME). Tumors generally tend to be naturally infiltrated with effector lymphocytes and are considered to be “immunogenic” neoplasms. However, instead of being infiltrated with large numbers of effector lymphocytes, PDAC is characterized by a highly immunosuppressive TME, which comprises cancer cells as well as immunosuppressive regulatory cells including cancer-associated fibroblasts,[9] tolerogenic dendritic cells,[10] myeloid-derived suppression cells,[11] tumor-associated macrophages,[12] and regulatory T cells.[13] As a result, PDACs are generally considered to be “non-immunogenic” neoplasms and show weak antitumor responses induced by immune-based therapies such as vaccines. The accumulation of these immunosuppressive regulatory cells in PDACs is likely to be closely related to the disease extent and provides one potential explanation for the limited effectiveness of immunotherapies for the treatment of pancreatic cancer.[14] As the predominant natural Ab found in humans, anti-Gal accounts for approximately 1% of immunoglobulins. Moreover, anti-Gal is known to be produced by both apes and Old World monkeys.[15] The ligand of anti-Gal, “α-gal epitope,” is a carbohydrate antigen with the structure Galα1-3Galβ1-4GlcNAc-R. The α-gal epitope is expressed as a major carbohydrate antigen by non-primate mammals, prosimians, and New World monkeys. Anti-Gal is known to play a role in the pathogenesis of some immunological conditions. Specifically, in pig xenografts, anti-Gal IgM and IgG mediate graft rejection through the abundant expression of α-gal epitopes.[16] Thus, it appears possible to exploit anti-Gal abundance to achieve good clinical outcomes. One potentially useful application is the enhancement of the immunogenicity of tumor-associated antigens (TAAs) through the development of vaccines that promote effective uptake by antigen-presenting cells (APCs), such as dendritic cells and macrophages.[16,17] Namely, TAAs on PDAC cells do not express markers that contain modifications recognized by APCs. To increase the immunogenicity of TAAs against APCs, IgG bound to TAAs could be a suitable strategy. APCs express Fcγ receptors (FcγR) (e.g., Fcγ RI/CD64, Fcγ RII/CD32, Fcγ RIII/CD16). These FcγR can effectively bind and mediate the internalization of opsonized cells (i.e., cells with bound IgG molecules), cell membranes, or TAAs via the Fc portion of the opsonizing IgG. This results in effective enhancement of the immunogenicity of the antigen complexed with IgG. Therefore, vaccination of cancer patients with whole tumor cell vaccines modified to express α-gal epitopes, should result in *in situ* binding of the patient’s anti-Gal IgG molecules to α-gal epitopes on the vaccinating cell membrane.

In our previous study, we demonstrated the *in vitro* and *in vivo* effectiveness of immunotherapy through vaccination, with a resultant increase in immunogenicity of α-gal Mucin 1 (MUC1). Furthermore, we showed that repeated vaccination with α-gal PANC-1 whole-cell vaccine stimulated the production of anti-MUC1 Ab, as well as the generation of an effective cytotoxic T lymphocyte response toward the MUC1 molecule.[18] However, the elicited antitumor immune response was relatively weak. To develop a more effective vaccine-based immunotherapy for PDAC, we hypothesized that resected tumor tissue lysates from patients might be an attractive source of PDAC-associated antigens for vaccination. These lysates contain several known antigens, such as MUC1 and mesothelin, as well as a spectrum of unidentified antigens expressed in cancer and stromal cells, potentially eliciting a broad range of anti-PDAC immune responses.[19] Accordingly, the generation of cancer vaccines from human PDAC tumor lysates engineered to express α-gal epitopes could enhance the immunogenicity of a broad spectrum of PDAC-associated antigens. MUC1 and mesothelin, in particular, are PDAC-associated glycoprotein antigens that have several potential N-glycan sites that are targets for α1,3-galactosyltransferase (α1,3GT), an enzyme that biosynthesizes α-gal epitopes on carbohydrates of PDAC-associated antigens (MUC1: 5 potential sites,[20] mesothelin: 4 potential sites[21]). MUC1 and mesothelin can effectively bind natural anti-Gal Ab *in situ* at the vaccination site, resulting in efficient recognition by APCs according to the mechanism described above.[20–22]

A major obstacle in the preparation of tumor lysate vaccines for clinical application is the isolation of sufficient numbers of live PDAC cells. We addressed this issue by preparing tumor lysates from PDAC tumors freshly resected from patients, thereby providing an alternative source of PDAC-associated antigens. The present study presents a novel immunotherapy expressing α-gal epitopes using freshly obtained human PDAC tumor tissue homogenates from patients and provides a mechanism by which autologous PDAC tumor lysate vaccines may target APCs *in situ* using a natural anti-Gal Ab. This approach could be applied to induce an effective antitumor immune response for the treatment of aggressive diseases such as PDAC.

## Materials and methods

### Ethics statement

All patients provided written informed consent for the use of tumor and normal pancreatic tissue for research purposes, and written consent was recorded in the patients’ electronic health records. The study was approved by the Institutional Review Boards of both hospitals (No. 550–5 for Osaka University, No. 23–29 for Kure Medical Center).

All animals were bred and maintained under specific pathogen-free conditions at the Institute of Experimental Animal Sciences, Osaka University Medical School. All animal care protocols and procedures described herein were approved by the Ethics Review Committee for Animal Experimentation of Osaka University (No. 25-045-1) and performed in accordance with the guidelines for the proper conduct of animal experiments from the Scientific Council of Japan. All surgery was performed under isoflurane anesthesia, and all efforts were made to minimize suffering.

### Patients

Tumor specimens were obtained from 10 patients at the time of surgical exploration for primary PDAC at Osaka University Hospital or the National Hospital Organization Kure Medical Center in 2012–2013. Patients with resectable cytologically or histologically proven PDACs were prospectively enrolled. Tumors and normal pancreatic tissues were transferred under sterile conditions from the operating room to the laboratory, where they were immediately frozen at -80°C. The tumor weights ranged between 100 and 700 mg.

### Mice

The mice used in this study had a disrupted *α1*,*3-galactosyltransferase* (*α1*,*3GT*) gene, and are hereafter referred to as α1,3GT knockout (KO) mice. Prior to the experimental procedures, anti-Gal Ab production was elicited in 6- to 8-week-old α1,3GT KO mice by four weekly intraperitoneal (i.p.) injections with 100 mg of pig kidney membrane homogenate.[23] These mice are hereafter referred to as high anti-Gal KO mice (i.e., α1,3GT KO mice injected with pig kidney fragments to induce high levels of anti-Gal Abs). The amount (titer) of anti-Gal Ab was confirmed to be similar to that observed in humans (1:400–1:2,000) by enzyme-linked immunosorbent assay (ELISA) as described previously.[18,19,23,24]

### Preparation of tumor lysate vaccines expressing α-gal epitopes

To synthesize α-gal epitopes on either tumor membranes or normal pancreatic tissue membranes, we employed recombinant (r) α1,3GT (a generous gift from Prof. Uri Galili, University of Massachusetts Medical School, MA, USA). The detailed methods of both α1,3GT enzyme isolation and synthesis of α-gal epitopes on the carbohydrate chains were described previously.[25,26]

To enzymatically synthesize α-gal epitopes on either PDAC membranes or normal pancreatic tissue, tissues were thawed, homogenized under sterile conditions, and washed two times in 100 ml of cold saline by centrifugation at 30,000 × *g*. The membranes were resuspended at 100 mg/ml in enzyme reaction buffer containing 0.1 M methylethylmorpholine sulfate (pH 6.2), 25 mM MnCl_2_, and 1 mM UDP-Gal (Merck Millipore, Germany). Subsequently, the membrane suspensions were incubated with both neuraminidase (1 mU/ml; Sigma-Aldrich, MO, USA) and rα1,3GT (50 μg/ml) with constant rotation for 15 h at 37°C. After incubation, the membranes were washed twice with saline containing 1 mM EDTA and twice with saline. Subsequently, the processed tumor membranes were resuspended at 100 mg/ml in saline, irradiated at 50 Gy, and frozen at -80°C until use.

### Western blot analysis of α-gal epitope expression on membrane glycoproteins

Twenty micrograms of processed or unprocessed membranes, including pig kidney membranes as a positive control, were subjected to 10% SDS-PAGE and then transferred electrophoretically onto a polyvinylidene difluoride membrane using a semi-dry electroblotting system.[18] The membrane was blocked overnight at 4°C with Blocking One (Nacalai Tesque, Japan), and then incubated with M86 anti-Gal mAb[18] (1:2 dilution) in phosphate-buffered saline (PBS) containing 1% bovine serum albumin (BSA) for 2 h at room temperature. After washing, the blots were incubated for 1 h with horseradish peroxidase (HRP)-conjugated goat anti-mouse IgM (1:1000 dilution) secondary Ab. The color reaction was developed using an ECL detection system (GE Healthcare, UK).

### Human PDAC tumor lysate or normal pancreatic tissue lysate vaccination

Twenty of the high anti-Gal KO mice described above were vaccinated by i.p. injection five times at 1-week intervals with 10 mg of irradiated unprocessed control (n = 10) or processed PDAC tumor lysate (n = 10) (hereafter referred to as α-gal(-) PDAC-ly or α-gal(+) PDAC-ly, respectively). To address whether uptake of anti-Gal opsonized tumor lysate by APCs induces an immune response against normal cell antigens, such as benign stromal cells, endothelial cells, or endocrine cells, 20 of the high anti-Gal KO mice were vaccinated with unprocessed normal (n = 10) or processed normal pancreatic tissue lysate (n = 10) obtained from resected pancreatic specimens (hereafter referred to as α-gal(-) N-ly or α-gal(+) N-ly, respectively), in a similar manner to α-gal tumor lysate vaccination. This experiment was undertaken to control for any side effects of α-gal(+) PDAC-ly vaccination. One week after the fifth vaccination, the mice were assessed for either antitumor or autoimmune responses induced by tumor lysate vaccination.

### Enzyme-linked immunosorbent assay (ELISA)

ELISA was performed to assess the production of anti-PDAC IgG in response to vaccination. Human PDAC cell lines, including PANC-1, MIAPaCa-2, and BxPC-3, were purchased from ATCC (Manassas, VA, USA). The cell lines were adjusted to 2 × 10^6^ cells/ml in PBS and plated in 50 μl aliquots in 96-well microtiter plates (IWAMKI, Japan). The plates were dried for 24 h to allow the cells to firmly attach to the wells. Subsequently, the plates were blocked with 1% BSA in carbonate buffer. Serum samples obtained from vaccinated α1,3GT KO mice were added at various dilutions to the wells in aliquots of 50 μl of PBS containing 1% BSA. After 2-h incubation at room temperature, the plates were washed in PBS containing 0.05% Tween 20 (Sigma-Aldrich) and then HRP-conjugated goat anti-mouse IgG was added to the wells (1:500 dilution; Bethyl Laboratories, Inc., TX, USA). The plates were further incubated for 1 h at room temperature and washed in PBS containing 0.05% Tween 20. The color reaction was developed with O-phenylenediamine dihydrochloride (Sigma-Aldrich) and absorbance was measured at 492 nm. To further evaluate the production of anti-MUC1 IgG and anti-mesothelin IgG, ELISA using either MUC1-BSA[18] or purified human mesothelin (Cat. No. H00010232; Abnova, Taiwan) as the solid-phase antigen was performed as previously described.[18,19]

### Enzyme-linked immunospot assay (ELISPOT)

An ELISPOT assay was employed to investigate the expansion of either anti-MUC1 secreting B cells, anti-mesothelin secreting B cells, MUC1-specific activated T cells, or mesothelin-specific activated T cells (i.e., IFN-γ secreting T cells), using a previously described method.[18,19] Furthermore, to investigate whether MUC1- and mesothelin-specific CD8^+^ T cells were generated in the spleen of KO mice vaccinated with α-gal(+) PDAC-ly, *in vitro* and *in vivo* depletion of CD8^+^ T cells in the ELISPOT assay was performed. For CD8^+^ T cell blockade *in vitro*, high anti-Gal KO mice (n = 10) were generated and then vaccinated with either α-gal(-) (n = 5) or α-gal(+) PDAC-ly (n = 5), respectively, in a similar manner as described above in the section describing PDAC tumor lysate vaccination. One week after the last vaccination, splenocytes were prepared from successfully vaccinated KO mice and incubated in ELISPOT wells with anti-CD8 mAb (Lyt 3.2) (clone 53–5.8, Cat. No. BE0223; Bio X Cell, NH, USA) at 10 μg/ml for blockade in the presence of either MUC1 or mesothelin peptide. After 24-h incubation at 37°C, the wells were washed and then stained for detection of MUC1- or mesothelin-specific activated T cells as previously described.[18,19] For CD8^+^ T cell depletion *in vivo*, high anti-Gal KO mice (n = 10) were generated and subsequently vaccinated with α-gal(+) PDAC-ly. Five vaccinated KO mice were simultaneously administered 300 μg of anti-CD8b mAb (Lyt 3.2) by i.p. injection at day -1 (one day before vaccination), day 0 (day of first vaccination), and every week after vaccinations with α-gal(+) PDAC-ly (days 8, 15, 22, and 29).[27,28] For controls, we employed the remaining five vaccinated KO mice. Equivalent doses of rat control Ig (clone 2A3, Cat. No. BE0089; Bio X Cell) were given i.p. to vaccinated KO mice in a similar manner as described above (n = 5). One week after the last mAb injection, splenocytes were prepared from successfully vaccinated and CD8^+^ T cell depleted KO mice, and ELISPOT assay for either MUC1- or mesothelin-specific activated T cells was performed in a similar manner as previously described.[18,19]

### *In vivo* studies of tumor lysate vaccination

Eighty high anti-Gal KO mice were generated by immunization with pig kidney fragments and subsequently vaccinated with unprocessed control (n = 20), processed PDAC tumor lysate (n = 20), and unprocessed normal (n = 20) or processed normal pancreatic tissue lysate (n = 20). One week after the last vaccination in a series of five vaccinations, splenocytes were prepared from successfully vaccinated donor KO mice and suspended in warm (37°C) sterile RPMI complete medium containing 50 μM 2-mercaptoethanol. For adoptive transfer, splenocytes were transferred by i.p. injection into NOD/SCID mice three times at 3-day intervals (75–150 × 10^6^ cells/vaccinated KO mouse). Splenocytes obtained from KO mice vaccinated with PDAC tumor lysates [α-gal(-) PDAC-ly (n = 5) or α-gal(+) PDAC-ly (n = 5)] or normal pancreatic tissue lysates [α-gal(-) N-ly (n = 5) or α-gal(+) N-ly (n = 5)] injected in equal amounts into NOD/SCID recipient mice (in total, 90 × 10^6^ splenocytes were transferred; each group, n = 5). One day after the last adoptive transfer, all NOD/SCID mice were challenged by subcutaneous (s.c.) injection with 10 × 10^6^ live PANC-1 cells. Subsequently, these mice were examined for tumor growth and survival. The general health of all mice was monitored daily after injection. Mice were sacrificed at the following defined humane endpoints: 1) physical appearance (self-injury, soiling of hair with urine or feces, bleeding, severe body weight loss as defined by > 20% loss in maximal body weight, loss of appetite), and 2) clinical physiology (tachypnea, low body temperature). Mice were humanely sacrificed when an increase in tumor size, defined as > 10% increase in body weight, was detected. Mice were anesthetized using isoflurane and subsequently sacrificed by cervical dislocation.

### Immunohistochemical analysis

To analyze MUC1 and mesothelin expression in resected PDAC tissue, tumor specimens were cut into small blocks, formalin-fixed, and embedded in paraffin. Briefly, formalin-fixed, paraffin-embedded 4-μm sections were deparaffinized in xylene. Next, antigen retrieval was performed with heat-induced epitope retrieval at 95°C for 40 min, and then the samples were incubated in methanol containing 0.3% hydrogen peroxide to block endogenous peroxidase. Following incubation with normal protein blocking serum (Vectastain Elite ABC kit; Vector Laboratories, Inc., CA, USA), the sections were incubated overnight at 4°C with either mouse anti-human MUC1 mAb (clone VU4H5, Cat. No. sc-7313, 1:100; Santa Cruz Biotechnology, Inc., TX, USA) or mouse anti-human mesothelin mAb (clone 5B2, Cat. No. LS-C87690, 1:20; LifeSpan BioSciences, Inc., WA, USA) as the primary Ab. Thereafter, the staining was developed with avidin-biotin complex reagents (Vector Laboratories, Inc.) using 3,3’-diaminobenzidine. All sections were counterstained with hematoxylin (Sigma-Aldrich).

To identify the phenotypes of infiltrating lymphocytes in PDAC tumors generated by the NOD/SCID tumor challenge model, tumor specimens were resected when they reached 50 mm^2^ in cross-sectional area, cut into small blocks, formalin-fixed, and embedded in paraffin. Tissue sections were incubated overnight at 4°C with either rat anti-mouse CD4 mAb (Cat. No. 103-M105, 1:500; ReliaTech GmbH, Germany) for CD4^+^ T cells or rat anti-mouse CD8 mAb (clone 53–6.7, Cat. No. 100701, 1:500; BioLegend, Inc., CA, USA) for CD8^+^ T cells. Staining was developed with avidin-biotin complex reagents using 3,3’-diaminobenzidine. All sections were counterstained with hematoxylin.

### Statistical analysis

Data were collected from at least five independent experiments. Quantitative data are expressed as the mean ± SD. Statistical analysis was performed using Student’s *t*-test or the Mann-Whitney U test. Kaplan-Meier curves of estimated survival were generated, and comparisons between each group were performed using a two-sided log-rank test. A *P*-value of < 0.05 was considered significant.

## Results

### Patient characteristics

The clinicopathological characteristics of the 10 patients enrolled and evaluated in this study are presented in Table 1. The patient group comprised 7 males and 3 females with a median age of 71 years (range, 53–80 years). Pancreatic cancers were located in the pancreatic head in 4 patients and body and tail in 6 patients. Three patients were diagnosed as UICC TNM stage III, six as stage IIB, and one as stage IA. Six patients underwent surgery without preoperative therapy, while four patients completed neoadjuvant chemoradiotherapy (NACRT). The NACRT treatment schedule was as follows: 50.4 Gy (1.8 Gy/day, 5 times per week, 28 fractions total of preoperative radiation) and administered concurrently with 30-min intravenous infusion of gemcitabine on days 1, 8, 22, and 29 or S-1 orally on days 1–5, 8–12, 22–26 and 29–33. Surgical exploration was conducted 4–7 weeks after the final dose of radiation.[29] The pathological response was defined as the fraction of degenerated cancer cells according to Evans classification.[30] The pathological response was judged as grade I in 2 patients, grade IIa in 1 patient, and grade IIb in 1 patient.

**Table 1:** Patient characteristics.

Clinicopathological characteristics	
Number of patients	10
Age (years; median, range)	71.5, 53–80
Gender (male/female)	7/3
Tumor location (head/body and tail)	4/6
Stage^a^ (IA/IB/IIA/IIB/III/IV)	1/0/6/3/0
Neoadjuvant CRT^b^ (+/−)	4/6
Response evaluation of neoadjuvant CRT = Evans classification (I/IIa/IIb/III/IV)	2/1/1/0/0
Histological classification^c^ (tub1/tub2/por)	0/9/1
Cancer stromal volume (medullary type/intermediate type/scirrhous type)	1/1/8
Tumor infiltrative (INF) pattern^d^ (INFα/INFβ/INFγ)	0/9/1
MUC1 expression (+/−)	10/0
Mesothelin expression (+/−)	10/0

^a^Stage; UICC 7^th^ edition

^b^CRT: chemoradiotherapy

^c^tub1: well differentiated tubular adenocarcinoma; tub2: moderately differentiated tubular adenocarcinoma; por: poorly differentiated adenocarcinoma

^d^Tumor infiltrative (INF) pattern: INFα; Tumor displays expanding growth with a distinct border from the surrounding tissue, INFβ; Tumor shows an intermediate pattern between INFα and INFγ, INFγ; Tumor displays infiltrative growth with no distinct border with the surrounding tissue.

PDAC TAAs, including MUC1 and mesothelin, are glycoproteins.[31,32] As shown in our previous studies,[18,19] α-gal epitopes can be biosynthesized by α1,3GT on the carbohydrates of these TAAs. Therefore, MUC1 and mesothelin can be presented to T cells by MHC class I and class II pathways, ultimately resulting in polyclonal expansion of both B and T cells. MUC1 and mesothelin were clearly expressed in PDAC tumors of all patients (Table 1, microscopic findings not shown).

### Synthesis of α-gal epitopes on either PDAC tumor lysates or normal pancreatic tissue lysates

**Fig 1:**
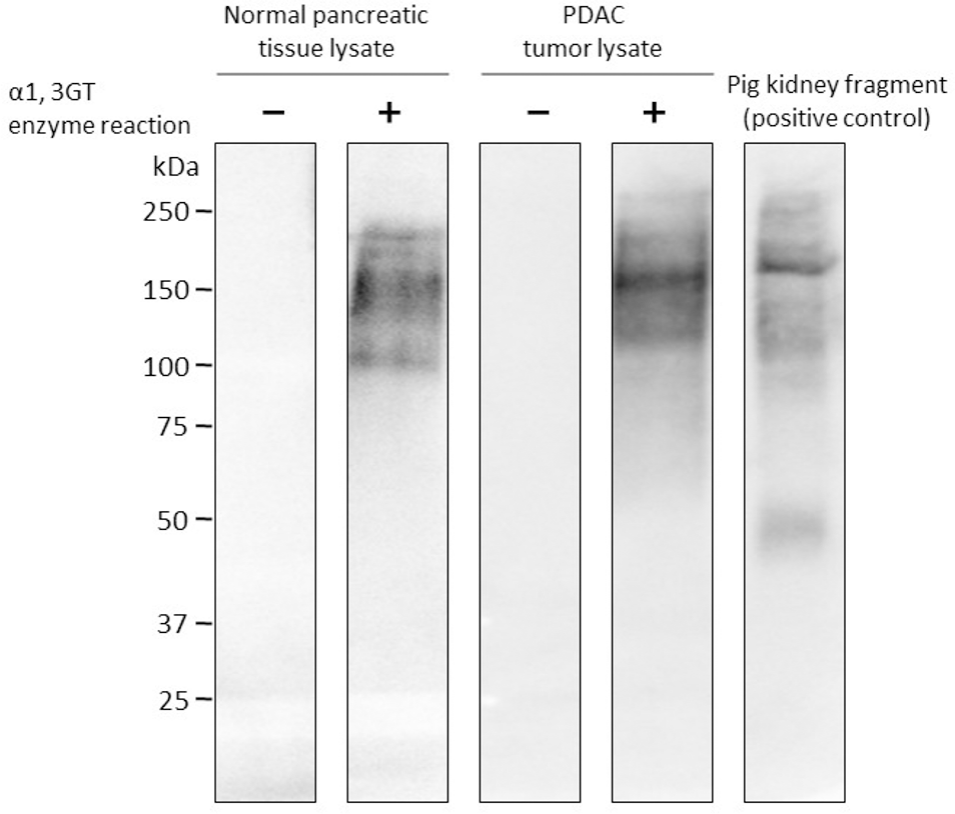
Biosynthesis of α-gal epitopes on pancreatic ductal adenocarcinoma (PDAC) tumor lysates and normal pancreatic tissue lysates assessed by Western blot analysis. Pig kidney membrane was employed as the positive control for α-gal epitope expression. High expression of α-gal epitopes was observed in both processed normal tissue and PDAC tumor lysates. No expression of α-gal epitopes was detected in both unprocessed normal tissue and PDAC tumor lysates.

PDAC tumor lysates and normal pancreatic tissue lysates obtained from 10 patients were assessed for the synthesis of α-gal epitopes by Western blot analysis. Representative data are shown in Fig 1. Pig kidney membrane was employed as the positive control for α-gal epitope expression. No expression of α-gal epitopes was observed on either control membrane [α-gal(-) N-ly and α-gal(-) PDAC-ly], which were not processed with α1,3GT, whereas high expression of α-gal epitopes was observed on both processed membranes [α-gal(+) N-ly and α-gal(+) PDAC-ly]. The staining patterns revealed large numbers of glycoprotein molecules of different sizes in either normal pancreatic tissue or PDAC lysates, which had been subjected to rα1,3GT processing, indicating that multiple α-gal epitopes had been added to their carbohydrate chains. Pig kidney membrane naturally expressed an abundance of α-gal epitopes, as indicated by the extensive binding of M86 anti-Gal mAb.

### Vaccination with α-gal PDAC-ly specifically elicits the production of either anti-PDAC cell Abs, anti-MUC1 Abs, or anti-mesothelin Abs

**Fig 2:**
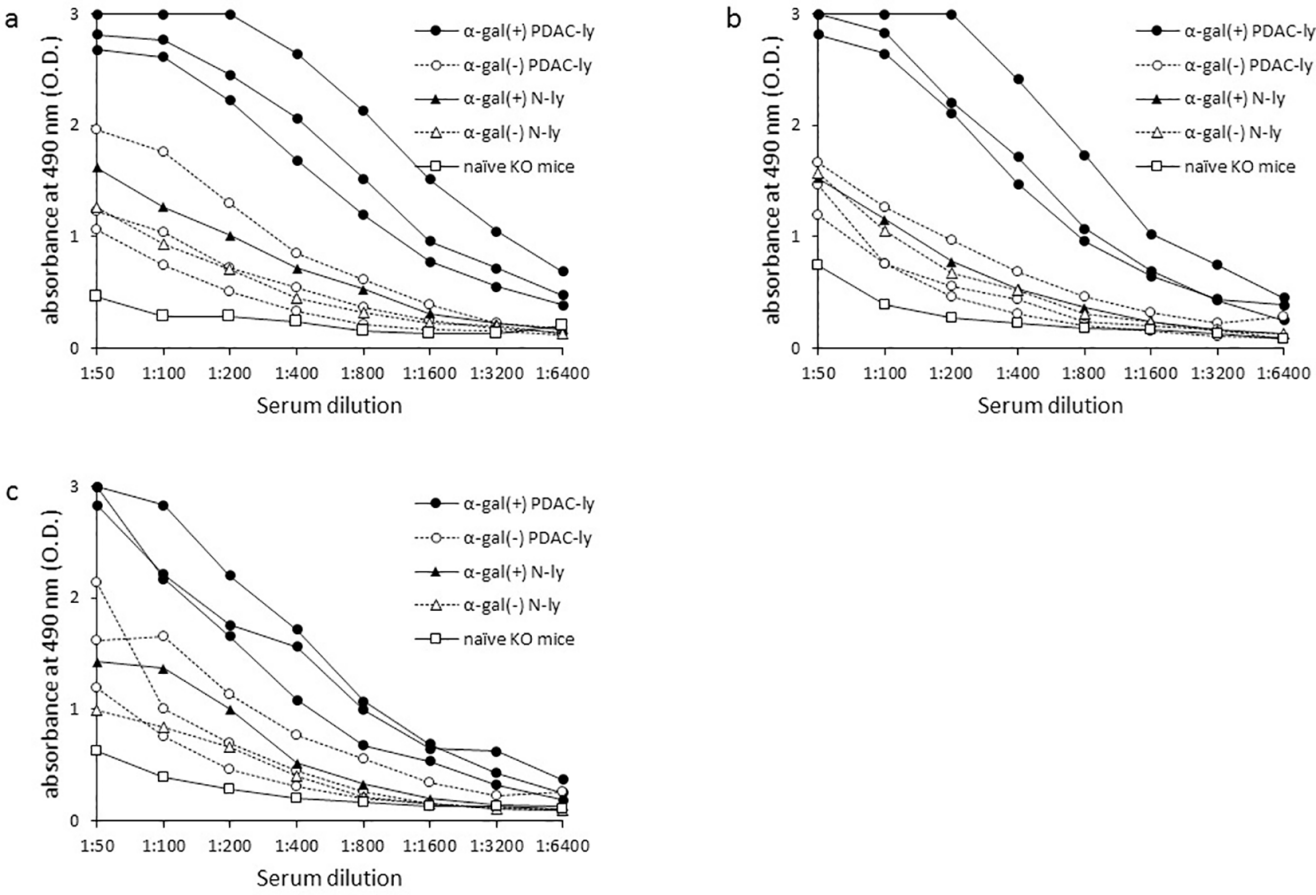
ELISA analysis of anti-PDAC cell IgG production induced by tumor lysate vaccination. (a) Anti-PANC-1 IgG production, (b) anti-MIAPaCa-2 IgG production, (c) anti-BxPC-3 IgG production. Vaccination with α-gal(+) PDAC-ly resulted in marked increase in the production of anti-PDAC cell IgG compared with α-gal(-) PDAC-ly vaccination. Vaccinations with either α-gal(-) or α-gal(+) N-ly did not elicit a significant anti-PDAC cell IgG response. Representative data from ten experiments with similar results are shown. ELISA results represent one or three data sets from n = 10 mice/group (one data set: naïve KO mice, α-gal(-) or α-gal(+) N-ly; three data sets: α-gal(-) or α-gal(+) PDAC-ly).

As shown in Fig 2A–2C, vaccination with α-gal(+) PDAC-ly resulted in a 16- to 32-fold increase in the production of anti-PANC-1, anti-MIAPaCa-2 or anti-BxPC-3 IgG compared with vaccination with α-gal(-) PDAC-ly. In contrast, five vaccinations with either α-gal(-) N-ly or α-gal(+) N-ly did not elicit a significant anti-PDAC cell IgG response. Although we predicted that vaccination with α-gal(-) PDAC-ly would also induce a low level of Ab production against PDAC cells, this was not observed, and the Ab response from α-gal(-) PDAC-ly vaccination was similar to that of either α-gal(-) N-ly or α-gal(+) N-ly vaccination.

**Fig 3:**
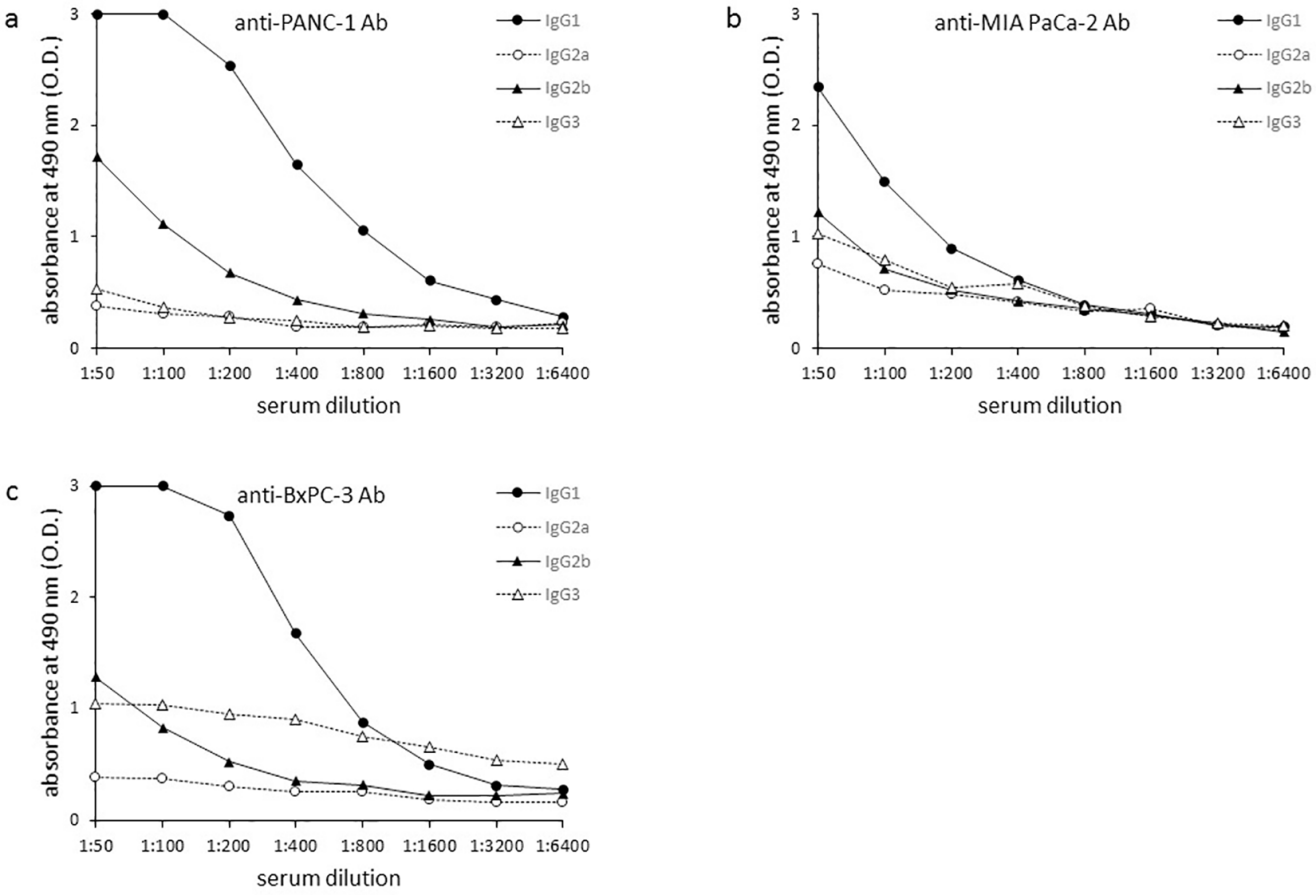
Anti-PDAC cell IgG subclasses production induced by tumor lysate vaccination. (a) Induced anti-PANC-1 IgG subclasses. (b) Induced anti-MIAPaCa-2 IgG subclasses. (c) Induced anti-BxPC-3 IgG subclasses. Representative data from five experiments with similar results are shown. ELISA results represent one data set from a group of 10 vaccinated mice.

Analysis of the IgG Ab subclasses elicited during anti-PDAC cell responses in α-gal(+) PDAC-ly vaccinated mice revealed a large amount of the IgG1 subclass, but no IgG2a, IgG2b, and IgG3 subclasses (Fig 3A–3C). Conversely, a similar analysis of IgG subclasses in α-gal(-) PDAC-ly vaccinated mice showed small amounts of IgG1 production, but no IgG2a, IgG2b, and IgG3 subclasses (data not shown).

**Fig 4:**
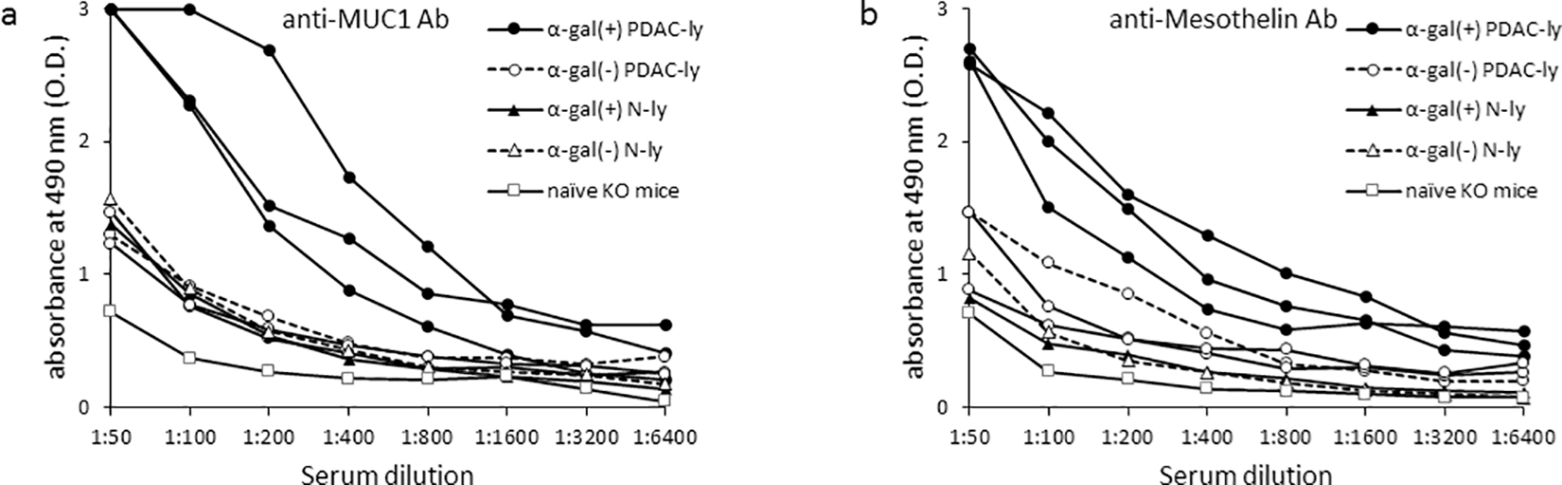
Anti-MUC1 IgG and anti-mesothelin IgG production induced by tumor lysate vaccination. (a) ELISA for anti-MUC1 IgG production. (b) ELISA for anti-mesothelin IgG production. Representative data from ten experiments with similar results are shown. ELISA results represent one or three data sets from n = 10 mice/group (one data set: naïve KO mice, α-gal(-) or α-gal(+) N-ly; three data sets: α-gal(-) or α-gal(+) PDAC-ly).

**Fig 5:**
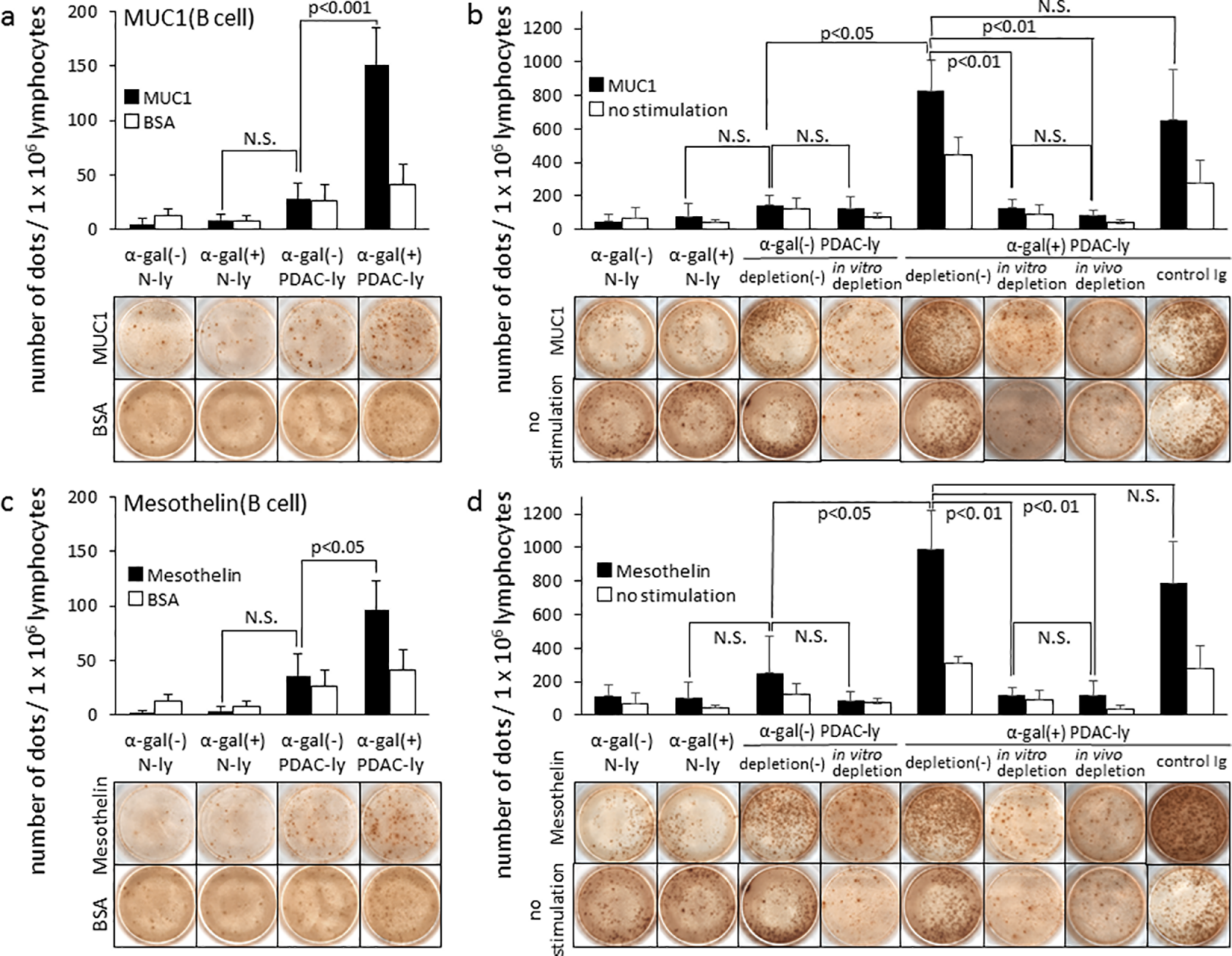
B cell and T cell expansion in response to tumor lysate vaccination. ELISPOT assays for (a) anti-MUC1 Ab secreting B cells and (b) MUC1-specific activated T cells and *in vitro* and *in vivo* depletion of CD8^+^ T cells, detected as IFN-γ secreting lymphocytes. ELISPOT assays for (c) anti-mesothelin Ab secreting B cells and (d) mesothelin-specific activated T cells and *in vitro* and *in vivo* depletion of CD8^+^ T cells, detected as IFN-γ secreting lymphocytes. Data represent the mean ± SD of five independent splenocyte preparations; bars, SD. Statistical analyses were performed using Student’s *t*-test. N.S.: not significant.

As shown in Fig 4A and 4B, vaccination with α-gal(+) PDAC-ly resulted in an 8-fold increase in the production of both anti-MUC1 IgG and anti-mesothelin IgG compared with α-gal(-) PDAC-ly vaccination. ELISAs did not detect significant Ab production against these TAAs following vaccination with α-gal(-) PDAC-ly, whereas α-gal(-) PDAC-ly vaccinated KO mice displayed a small increase in both anti-MUC1- and anti-mesothelin-Ab-secreting B cells assessed by ELISPOT assay compared with KO mice vaccinated with α-gal(-) N-ly or α-gal(+) N-ly (Fig 5A and 5C). No significant Ab responses against these TAA molecules were observed in either α-gal(-) N-ly or α-gal(+) N-ly vaccinated mice, and there were no significant differences in Ab responses between α-gal(-) N-ly or α-gal(+) N-ly vaccination and α-gal(-) PDAC-ly vaccination (Fig 4A and 4B, Fig 5A and 5C).

### Vaccination with α-gal(+) PDAC-ly induces an effective antitumor immune response from B and T cells against both MUC1 and mesothelin

As shown in Fig 4B and 4E, splenocytes isolated from α-gal(-) PDAC-ly vaccinated KO mice showed 28.7 ± 13.3 spots/1 × 10^6^ splenocytes of anti-MUC1 secreting B cells and 36.5 ± 19.3 spots/1 × 10^6^ splenocytes of anti-mesothelin secreting B cells. In comparison, α-gal(+) PDAC-ly vaccinated KO mice showed 151.8 ± 33.3 spots/1 × 10^6^ splenocytes of anti-MUC1 secreting B cells and 97.2 ± 26.0 spots/1 × 10^6^ splenocytes of anti-mesothelin secreting B cells. The numbers of ELISPOT spots of α-gal(+) PDAC-ly vaccinated KO mice were significantly greater than those of α-gal(-) PDAC-ly vaccinated KO mice (MUC1: *P* = 0.0008; mesothelin: *P* = 0.029). α-Gal(-) N-ly and α-gal(+) N-ly vaccinated KO mice did not display significant B cell spots in both MUC1 and mesothelin ELISPOT wells.

As shown in Fig 5B and 5D, the priming of T cells specifically responsive to either MUC1 or mesothelin peptide was determined using the ELISPOT assay to detect IFN-γ secretion following the *in vitro* activation of T cells in the presence of either MUC1 or mesothelin peptides. In α-gal(-) PDAC-ly vaccinated KO mice, 146.0 ± 59.0 and 123.3 ± 65.6 spots/1 × 10^6^ splenocytes were detected in the presence and absence of MUC1 peptide, respectively, with no significant increase in the number of spots observed (*P* = 0.81; Fig 5B). In similarly vaccinated KO mice, 250.7 ± 87.5 and 123.3 ± 65.6 spots/1 × 10^6^ splenocytes were detected in the presence and absence of mesothelin peptide, respectively, and no significant increase in the number of spots was observed (*P* = 0.31; Fig 5D). In α-gal(+) PDAC-ly vaccinated KO mice, 828.0 ± 180.5 and 312.0 ± 40.9 spots/1 × 10^6^ splenocytes were detected in the presence or absence of MUC1 peptide stimulation, respectively, and a significant difference in the number of spots was observed with and without MUC1 peptide (*P* = 0.027; Fig 5B). Furthermore, the number of spots in the presence of MUC1 peptide was significantly higher in the α-gal(+) PDAC-ly vaccinated group compared with the α-gal(-) PDAC-ly group (*P* = 0.049; Fig 5B). For mesothelin-specific T cell spots, 988.0 ± 232.5 and 312.0 ± 40.9 spots/1 × 10^6^ splenocytes were detected with or without mesothelin peptide in the α-gal(+) PDAC-ly group, respectively, indicating a significant difference (*P* = 0.033; Fig 5D). Moreover, the number of spots following stimulation with mesothelin peptide was significantly higher in the α-gal(+) PDAC-ly vaccinated group compared with the α-gal(-) PDAC-ly group (*P* = 0.021; Fig 5D).

In the analysis of CD8^+^ T cell depletion *in vitro* and *in vivo*, depletion in both situations with anti-CD8^+^ mAb significantly blocked the elicited increase in the number of spots for both MUC1- and mesothelin-specific T cells in α-gal(+) PDAC-ly vaccinated KO mice (Fig 5B and 5D). Conversely, control splenocytes from mice vaccinated with α-gal(-) PDAC-ly and treated *in vitro* with anti-CD8 mAb displayed a similar number of spots upon stimulation with either MUC1 or mesothelin peptides in comparison with those in α-gal(-) PDAC-ly vaccinated KO mice without anti-CD8 mAb treatment (Fig 5B and 5D). Similarly, control splenocytes from mice vaccinated with α-gal(+) PDAC-ly and subsequently injected with rat control Ig displayed a similar number of spots upon stimulation with these PDAC-associated peptides in comparison with those in α-gal(+) PDAC-ly vaccinated KO mice (Fig 5B and 5D). Splenocytes treated *in vitro* with anti-CD8 mAb displayed 129.3 ± 75.8 spots in the presence of MUC1 peptide stimulation (*P* = 0.0050) and 117.3 ± 46.5 spots in the presence of mesothelin peptide stimulation (*P* = 0.0054), respectively. Splenocytes treated *in vivo* with anti-CD8 mAb displayed 85.3 ± 30.8 spots in the presence of MUC1 peptide stimulation (*P* = 0.0037) and 120.0 ± 84.0 spots in the presence of mesothelin peptide stimulation (*P* = 0.0055), respectively. These numbers of spots were comparable with those in the absence of either MUC1 or mesothelin peptide stimulation, respectively (MUC1 *in vitro*: *P* = 0.82, MUC1 *in vivo*: *P* = 0.18, Fig 5B; mesothelin *in vitro*: *P* = 0.65, mesothelin *in vivo*: *P* = 0.27, Fig 5D). Taken together, MUC1- and mesothelin-specific CD8+ T cells were clearly generated in the spleen of KO mice vaccinated with α-gal(+) PDAC-ly as confirmed by i*n vitro* and *in vivo* CD8^+^ T cell depletion.

For the clinical application of immunotherapy with autologous tumor lysate vaccines engineered to express α-gal epitopes, the toxicity and safety of the injection of α-gal tumor lysates in humans must be assessed. One concern about autologous tumor lysate vaccines is the presence of normal antigens in the vaccine, either from tumor cells or normal cells, which may disrupt tolerance and induce an autoimmune response to normal antigens. This concern was addressed by evaluating high anti-Gal KO mice repeatedly vaccinated (five times) with 100 mg of syngeneic kidney, liver, or pancreas membranes processed to express α-gal epitopes. One week after the completion of the immunization protocol, autoAb production including anti-kidney, anti-liver, or anti-pancreas Abs was assessed by ELISA. No autoAbs were detected in the sera of these immunized KO mice. Furthermore, 3 months after immunization was completed, the kidneys, livers, and pancreas of the immunized KO mice were histologically analyzed. Autoimmune reactions within these organs were not detected, evidenced by normal tissue structure and the absence of inflammatory cell infiltrates. Further studies were also performed in high anti-Gal KO mice that were repeatedly immunized (five times) with 10 mg of unprocessed or processed human pancreatic tissue lysates, and assessed for autoimmune responses. This immunization regimen did not disrupt tolerance, as indicated by the absence of autoAbs and inflammatory processes in the pancreata of immunized mice, and normal kidney and liver function were observed, as assessed by blood examination. Taken together, these findings suggest that the ability of autologous tumor lysate vaccines expressing α-gal epitopes to stimulate autoimmune responses against normal tissue antigens in the vaccine is limited. Nevertheless, for clinical study, patients vaccinated with autologous α-gal tumor lysate vaccines should be closely monitored for autoimmune responses.

### Adoptive transfer of splenocytes from α-gal(+) PDAC-ly vaccinated KO mice protects and prolongs survival against tumor challenge using PANC-1 cells

**Fig 6:**
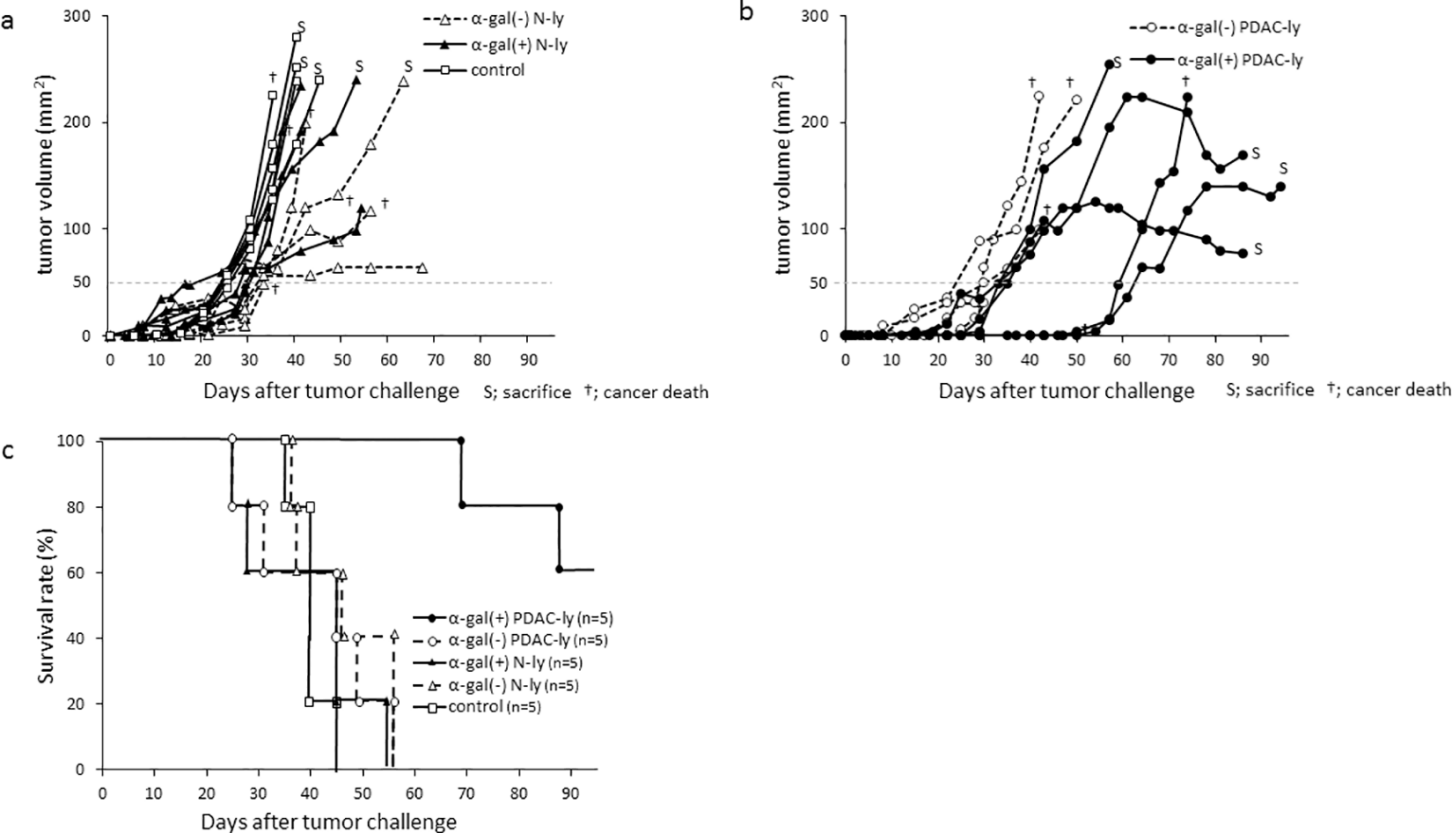
*In vivo* tumor growth and survival of adoptive transferred NOD/SCID mice challenged with live PANC-1 cells. (a) Size of subcutaneous tumors of NOD/SCID mice to which splenocytes were adoptively transferred from normal pancreatic tissue lysate vaccinated KO mice. □: α-gal(+) N-ly vaccinated KO mice, △: α-gal(-) N-ly vaccinated KO mice. The tumor sizes of five individual recipients in each group after adoptive transfer are shown. (b) Size of subcutaneous tumors of NOD/SCID mice to which splenocytes were adoptively transferred from PDAC tumor lysate vaccinated KO mice. ●: α-gal(+) PDAC-ly vaccinated KO mice, ○: α-gal(-) PDAC-ly vaccinated KO mice. The tumor sizes of five individual recipients in each group after adoptive transfer are shown. S: sacrifice, †: cancer death. (c) Survival curves of adoptive transferred NOD/SCID mice after tumor cell challenge with live PANC-1 cells. The curves were generated by the Kaplan-Meier method and assessed by the log-rank test.

*In vivo* tumor growth and representative images of NOD/SCID mice treated with either α-gal(-) N-ly, α-gal(+) N-ly, α-gal(-) PDAC-ly, or α-gal(+) PDAC-ly are shown in Fig 6A and 6B. NOD/SCID mice that received splenocytes from untreated control mice or α-gal(-) N-ly, α-gal(+) N-ly, and α-gal(-) PDAC-ly vaccinated KO mice developed large tumors, whereas α-gal(+) PDAC-ly vaccinated mice exhibited significantly slower tumor growth (Fig 6A and 6B). The *in vivo* results, including tumor growth rate and survival time, are summarized in Table 2. The untreated control, α-gal(-) N-ly, α-gal(+) N-ly, and α-gal(-) PDAC-ly groups showed no significant differences in the time to appearance of palpable tumors. In contrast, the α-gal(+) PDAC-ly group showed significantly delayed development of palpable tumors compared with the untreated, α-gal(-) N-ly, α-gal(+) N-ly, and α-gal(-) PDAC-ly groups. Tumors reached 50 mm^2^ in cross-sectional area within 25–30 days in the untreated group (mean, 26.9 ± 2.2 days), α-gal(-) N-ly group (mean, 31.5 ± 3.7 days), α-gal(+) N-ly group (mean, 25.7 ± 7.6 days), and α-gal(-) PDAC-ly group (mean, 28.7 ± 0.6 days). Tumors in NOD/SCID mice that received adoptive transfers from α-gal(+) PDAC-ly vaccinated mice grew to a similar size within 36–63 days (mean, 47.0 ± 14.7 days).

**Table 2:** The *in vivo* antitumor response against live PANC-1 cells in adoptive transferred NOD/SCID mice.

Type of vaccination	Untreated control mice (no vaccination) (n = 10)	Normal pancreatic tissue lysate		PDAC tumor lysate		*P* value vs. T(α-gal+)
		α-gal(-) (n = 5)	α-gal(+) (n = 5)	α-gal(-) (n = 5)	α-gal(+) (n = 5)	
Time to appearance of palpable tumor (days, Mean ± SD)	14.0 ± 2.2^a^	17.0 ± 9.7^b^	10.0 ± 5.5^c^	17.4 ± 6.9^d^	38.2 ± 16.6	a: *P*<0.01, b-d: *P*<0.05
Time to reach tumor size of 50 mm^2^ (days, Mean ± SD)	26.9 ± 2.2^e^	31.5 ± 3.7^f^	25.7 ± 7.6^g^	28.7 ± 0.6^h^	47.0 ± 14.7	e: *P*<0.01, f-h: *P*<0.05
Median survival time (days, 95%CI)	40.0 (35–45)^i^	46.0 (36–56)^j^	45.0 (25–55)^k^	45.0 (25–56)^l^	95.0 (69–95)	i-l: *P*<0.01

Data are presented as the mean ± SD from ten or five independent experiments.

Statistical analysis: a/e/i; untreated control mice vs.

α-gal(+) PDAC-ly

b/f/j; α-gal(-) N-ly vs. α-gal(+) PDAC-ly

c/g/k; α-gal(+) N-ly vs. α-gal(+) PDAC-ly

d/h/l; α-gal(-) PDAC-ly vs. α-gal(+) PDAC-ly

α-gal(+) PDAC-ly vaccines also prolonged survival after tumor challenge. As shown in Fig 6C and Table 2, the median survival time of NOD/SCID mice that received adoptive transfers from α-gal(+) PDAC-ly vaccinated KO mice was significantly extended (95.0 days, 95% CI 69–95 days) compared with the untreated control (40.0 days, 95% CI 35–45 days), α-gal(-) N-ly (46 days, 95% CI 36–56 days), α-gal(+) N-ly (45 days, 95% CI 25–55 days), and α-gal(-) PDAC-ly groups (45 days, 95% CI 25–56 days). The final cause of death for adoptive transferred NOD/SCID mice from untreated, α-gal(-) N-ly, α-gal(+) N-ly, α-gal(-) PDAC-ly, and α-gal(+) PDAC-ly groups was cancer death.

To assess unexpected adverse events, such as kidney and liver dysfunction, during vaccine treatment with either normal pancreatic tissue lysate or PDAC tumor lysate, blood examination, including creatinine, blood urea nitrogen, aspartate transaminase and alanine transaminase, was performed before/after vaccination (Table 3). No significant deterioration in kidney or liver function was detected after vaccine treatment.

**Table 3:** *In vivo* kidney and liver function before/after tumor lysate vaccination in high anti-Gal KO mice.

Factors	Naïve KO mice (before vaccination)	Normal pancreatic tissue lysate		PDAC tumor lysate		*P* value vs. T(α-gal+)
		α-gal(-)	α-gal(+)	α-gal(-)	α-gal(+)	
BUN	24.3 ± 4.9^a^	27.1 ± 3.3^b^	25.8 ± 3.8^c^	26.5 ± 1.9^d^	29.1 ± 5.4	N.S.
Cre	0.14 ± 0.00^e^	0.13 ± 0.02^f^	0.12 ± 0.2^g^	0.11 ± 0.03^h^	0.12 ± 0.02	N.S.
AST	171.5 ± 135^i^	152 ± 153^j^	93.4 ± 26.4^k^	140.8 ± 49.6^l^	134.3 ± 59.4	N.S.
ALT	33.0 ± 9.9^m^	37.0 ± 24.0^n^	27.0 ± 6.1^o^	33.6 ± 5.2^p^	36.9 ± 15.0	N.S.

Data are expressed as the mean ± SD from five independent experiments. N.S.: Not significant, BUN: blood urea nitrogen, Cre: creatinine, AST: aspartate transaminase, ALT: alanine transaminase

ALT: alanine transaminase. Statistical analysis

a/e/i/m; naïve KO mice vs. α-gal(+) PDAC-ly

b/f/j/n; α-gal(-) N-ly vs. α-gal(+) PDAC-ly

c/g/k/o; α-gal(+) N-ly vs. α-gal(+) PDAC-ly

d/h/l/p; α-gal(-) PDAC-ly vs. α-gal(+) PDAC-ly

### Vaccine treatment with α-gal(+) PDAC-ly induces extensive recruitment of CD4^+^ and CD8^+^ T cells into the challenged tumor lesion

**Fig 7:**
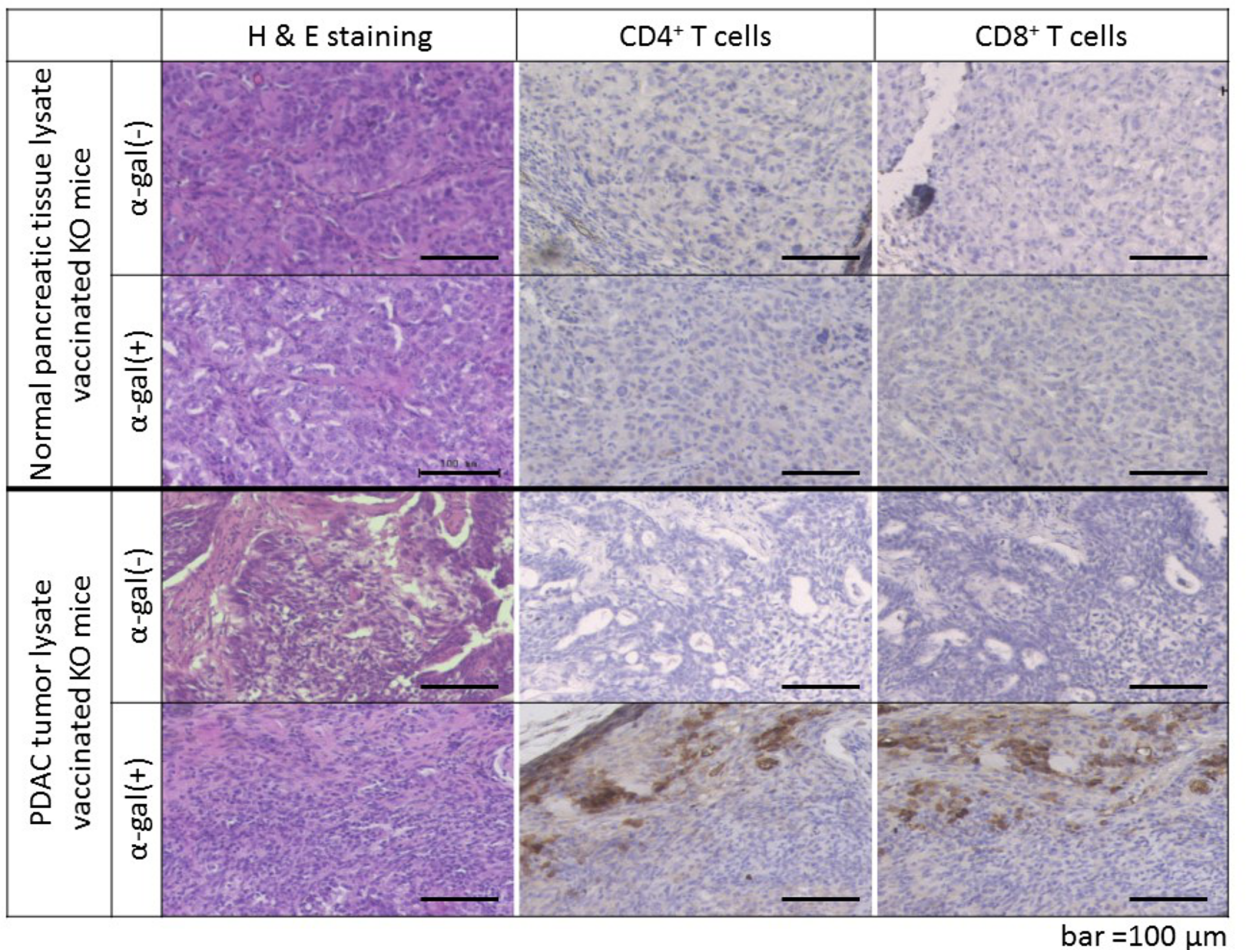
α-gal(+) PDAC-tumor lysate vaccination induced the infiltration of lymphocytes (CD4^+^ and CD8^+^ T cells and macrophages) into subcutaneous PANC-1 tumors. No infiltration of lymphocytes was observed in the α-gal(-) N-ly/α-gal(+) N-ly vaccinated groups and α-gal(-) PDAC-ly vaccinated group. However, infiltration by substantial numbers of both CD4^+^ and CD8^+^ T cells and macrophages was observed in the α-gal(+) PDAC-ly vaccinated group. H&E stained sections of the α-gal(+) PDAC-ly vaccinated group demonstrate severe destruction of PDAC cells elicited by effector cell infiltration. Representative images of five individual recipients in each group are shown. Scale bars = 100 µm.

To assess the antitumor properties of α-gal(+) PDAC-ly vaccination, we investigated the phenotypes of infiltrating lymphocytes, including CD4^+^ and CD8^+^ T cells and macrophages. Tumor lesions reaching a subcutaneous size of approximately 50 mm^2^ were resected at different time points (within 24–63 days post-s.c. inoculation of live PANC-1 cells) for histological analysis. No intratumoral infiltration of inflammatory cells was found in inoculated PANC-1 tumor lesions of α-gal(-) N-ly/α-gal(+) N-ly vaccinated KO mice or α-gal(-) PDAC-ly vaccinated KO mice (Fig 7). In comparison, extensive recruitment of CD4^+^ and CD8^+^ T cells and macrophages was detected in tumor lesions of α-gal(+) PDAC-ly vaccinated KO mice (Fig 7). Furthermore, strong disruption of PDAC cells in tumor lesions was observed in α-gal(+) PDAC-ly vaccinated KO mice, whereas a large number of viable PDAC cells was found in tumor lesions of α-gal(-) N-ly/α-gal(+) N-ly vaccinated KO mice and α-gal(-) PDAC-ly vaccinated KO mice. These histological results suggest that the marked prolongation of survival of mice treated with α-gal(+) PDAC-ly vaccination is attributable to the strong antitumor response induced by α-gal(+) PDAC-ly vaccine treatment.

### Vaccine treatment with α-gal(+) PDAC-ly originating from preoperatively treated tumor specimens effectively induces antibody production against PANC-1 cells and MUC1 peptide

A variety of preoperative combination therapies, including 5-FU and radiation, gemcitabine and radiation, or S-1 and radiation, have shown considerable effectiveness in the treatment of resectable PDAC.[30,33–35] Recently, we and others reported encouraging survival rates following gemcitabine-based NACRT in patients with potentially resectable PDAC.[36–38] Furthermore, we conducted NACRT consisting of gemcitabine and S-1 concurrent with full-dose radiation as a single-arm phase I trial.[29]

**Fig 8:**
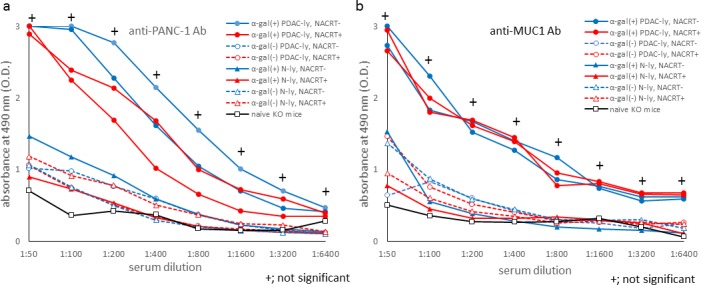
Antibody production against PANC-1 cells and MUC1 peptide induced by tumor lysates originating from patients treated with or without neoadjuvant chemoradiotherapy. (a) Anti-PANC-1 IgG production induced by tumor lysate vaccines generated from patients treated with or without NACRT. (b) Anti-MUC1 IgG production induced by tumor lysate vaccines generated from patients treated with or without NACRT. +: not significant between α-gal(+) PDAC-ly treated with NACRT vaccinated group and α-gal(+) PDAC-ly treated without NACRT vaccinated group. Representative data from five experiments with similar results are shown. ELISA results represent two data sets from n = 5 mice/group.

For the present study, we employed PDAC tumor specimens obtained from four patients treated with NACRT to prepare tumor lysate vaccines expressing α-gal epitopes. We found that the production of both anti-PANC-1 and anti-MUC1 Abs was effectively elicited by vaccination with α-gal(+) PDAC tumor lysates from patients treated with or without NACRT (Fig 8). Additionally, no significant differences were detected in the levels of these Abs in comparisons between α-gal(+) tumor lysates from patients treated with or without NACRT.

**Fig 9:**
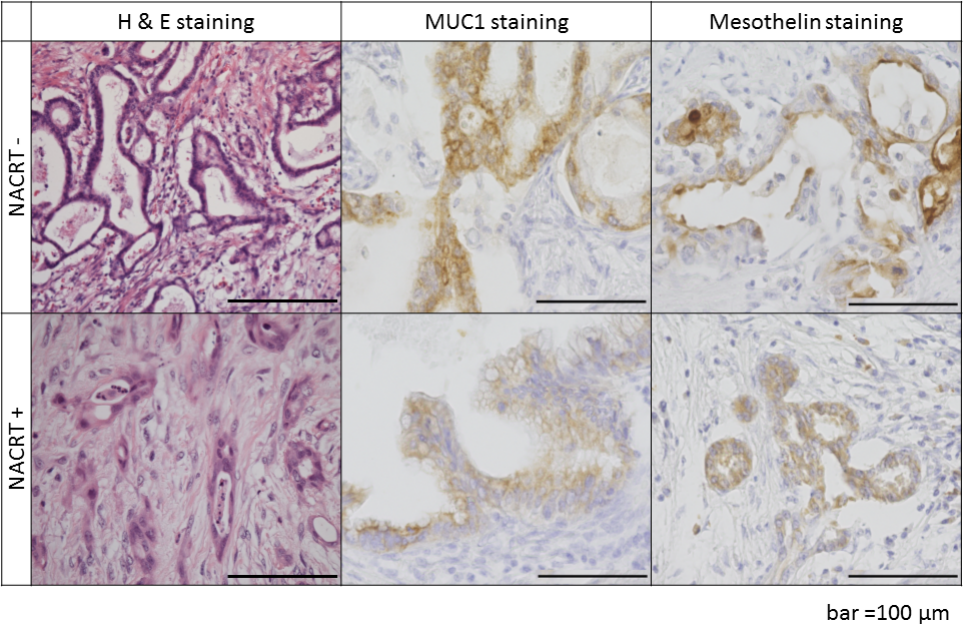
Immunohistological findings of original PDAC tumors obtained from patients treated with or without neoadjuvant chemoradiotherapy. H&E stained sections of PDAC tumors clearly demonstrate viable PDAC cells in the tumor treated without NACRT. However, grade IIa destruction of PDAC cells (Evans classification) was detected in the tumor treated with NACRT. Expression of MUC1 and mesothelin was observed in PDAC tumors treated with or without NACRT. The expression levels of these TAAs were similar between PDAC tumors treated with or without NACRT. Representative images of four individual patients are shown. Scale bars = 100 μm.

As shown in Fig 9, irradiation and chemotherapy against the original PDAC tumors did not affect the quality of the vaccinating materials, such as PDAC-associated antigens including MUC1 and mesothelin. Accordingly, α-gal(+) PDAC tumor lysates originating from preoperatively treated tumor specimens were also able to elicit a significant antitumor immune response.

## Discussion

Pancreatic cancer is generally lethal because remnant cells, including differentiated cancer cells and cancer stem cells remaining after surgery, chemotherapy, and radiation therapy, develop into recurrent or metastatic tumors. One approach to targeting these residual cancer cells is the vaccination-induced activation of immunocytes that can specifically attack and destroy TAA-expressing cancer cells. However, a major consideration in cancer immunotherapy is the TAA characteristics that generate effective vaccines. Overexpressed normal antigens[39–41] and viral antigens[42,43] are localized on various tumors, potentially acting as targets for immunotherapy. For most cancer types, particularly PDAC, no specific immunotherapy treatment is presently available to patients, and TAAs remain largely uncharacterized. In addition, antitumor immunity against PDAC-associated single antigens, such as MUC1 or mesothelin, may not be effective against heterogeneous tumor cell populations and carries the risk of inducing tumor antigen escape variants.[44,45] Therefore, effective tumor vaccines are generally acknowledged to require the inclusion of multiple TAAs that can be presented to T cells via MHC class I and class II pathways,[46–48] also known as polyvalent tumor lysate vaccines.[19]

The present study demonstrates a novel method for preparing human PDAC tumor lysate vaccines by processing PDAC tumor membranes to allow for their opsonization by an abundant (human) natural IgG Ab. Such *in situ* binding can target the vaccination tumor membranes to APCs, thereby increasing the uptake, processing, and presentation of PDAC-associated antigens to the immune system. This method exploits the synthesis of α-gal epitopes on the PDAC tumor membranes obtained from resected specimens by rα1,3GT and on the presence of natural anti-Gal Ab. To our knowledge, this is the first report to demonstrate that vaccination with α-gal PDAC tumor lysates originating from resected tumor specimens of patients elicited strong Ab production against human PDAC cells and MUC1 and mesothelin peptides, and led to effective activation of T cells specific to both MUC1 and mesothelin peptides. Furthermore, *in vivo* experiments with live human PDAC cells revealed significant slowing of tumor development in adoptive transferred NOD/SCID mice.

Western blot analysis of processed PDAC membranes with M86 anti-Gal mAb indicated that α-gal epitopes are produced on a variety of cell surface glycoproteins, which range in size from 50 to 200 kDa. This implies that rα1,3GT does not have selective activity and can synthesize α-gal epitopes on a broad spectrum of glycoproteins, including the PDAC-associated antigens MUC1 and mesothelin, which express N-acetyllactosamine residues on their carbohydrate chains. Importantly, unprocessed PDAC membranes do not bind M86 anti-Gal mAb. These findings indicate that similar binding of the patient’s anti-Gal occurs *in situ* at the vaccination site, thus targeting the vaccinating membranes for effective uptake by APCs. Since the α-gal epitopes are covalently linked to the PDAC membranes, they are expected to show high *in vivo* stability until uptake by APCs that bind to the opsonizing anti-Gal Abs.

Although we plan to primarily employ processed autologous tumor lysates as vaccines, resected PDACs are generally small and provide a limited amount of lysate. In addition, most PDACs are inoperable because patients present with incurable metastatic disease. To overcome these limitations, we propose employment of α-gal(+) whole-cell vaccines created from human PDAC cell lines, including PANC-1. In this study, we demonstrated the efficacy of α-gal(+) PDAC tumor lysate vaccines in an experimental model involving adoptive transferred NOD/SCID mice challenged with PANC-1. Importantly, α-gal(+) PDAC-associated antigens, including MUC1 and other unknown TAAs in PDAC tumor lysates, were shared with those expressed by PANC-1 cells and induced antitumor Abs by α-gal(+) PDAC tumor lysate vaccination that strongly cross-reacted with human PDAC cell lines. Conversely, anti-PDAC Abs elicited by α-gal(+) whole-cell vaccines can target cancer cells of PDAC patients. These findings suggest that α-gal(+) whole-cell vaccines may be therapeutically effective for the treatment of PDAC, including patients with partially resectable or unresectable disease.

The *in vitro* and *in vivo* data of the present study indicate that α-gal(+) PDAC tumor lysate vaccination can induce remarkable antitumor immunity. However, the key challenge for the future clinical application of this anticancer vaccine is the effective treatment of preexisting PDAC tumors (i.e., therapeutic setting) rather than prophylaxis, as described in this study. We show for the first time that when challenged with live PDAC (PANC-1) cells by s.c. injection, KO mice treated with human tumor lysate vaccines expressing α-gal epitopes originating from operative specimens of PDAC patients exhibited significant inhibition of tumor growth and increased survival.

Overall, our findings suggest that the use of tumor lysate vaccines originating from resected specimens and engineered to express α-gal epitopes will elicit an effective immune response against PDAC cells. We are optimistic that these vaccines will ultimately improve the prognosis for patients with pancreatic cancer, and a platform is now established for the design of clinical trials to address vaccine efficacy and safety.

## Supporting information

S1 Fig Original photographs of Western blots from which Fig 1 was derived. Blot A, Patient B, normal pancreatic tissue lysates (blue square); Blot B, Patient E, PDAC tumor lysates (red square); Blot B, pig kidney fragment (green square).
(TIF)
