# Supplementary figures and images for: A practical approach to pancreatic cancer immunotherapy using resected tumor lysate vaccines processed to express α-gal epitopes
Source: PLoS One. 2017 Oct 27;12(10):e0184901. doi: 10.1371/journal.pone.0184901 (PMC5659602; doi:10.1371/journal.pone.0184901)

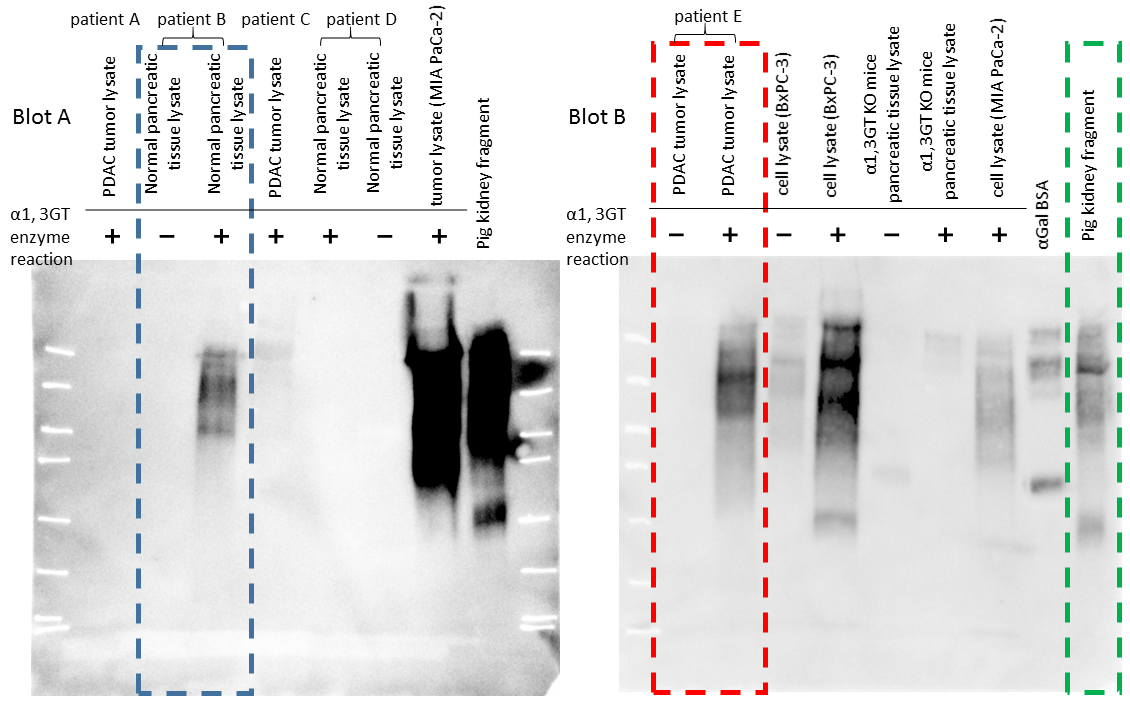

Supplement: S1 Fig — Blot A, Patient B, normal pancreatic tissue lysates (blue square); Blot B, Patient E, PDAC tumor lysates (red square); Blot B, pig kidney fragment (green square). (TIF) [file pone.0184901.s001.tif]
